# From osimertinib to preemptive combinations

**DOI:** 10.18632/oncotarget.28569

**Published:** 2024-03-15

**Authors:** Mikhail V. Blagosklonny

**Affiliations:** ^1^Roswell Park Comprehensive Cancer Center, Buffalo, NY 14263, USA

**Keywords:** lung cancer, NSCLC, EGFR, resistance, afatinib, gefitinib, capmatinib

## Abstract

Here, I suggest that while first-line osimertinib extends median progression-free survival (PFS) in EGFR-mutant lung cancer compared to first-generation TKIs, it reduces individual PFS in 15–20% of patients compared to first-generation TKIs. Since detecting a single resistant cell before treatment is usually impossible, osimertinib must be used in all patients as a first-line treatment, raising median PFS overall but harming some. The simplest remedy is a preemptive combination (PC) of osimertinib and gefitinib. A comprehensive PC (osimertinib, afatinib/gefitinib, and capmatinib) could dramatically increase PFS for 80% of patients compared to osimertinib alone, without harming anyone. This article also explores PCs for MET-driven lung cancer.

## INTRODUCTION

In EGFR-mutant-dependent non-small cell lung cancer (NSCLC), first- or second-generation EGFR-TKI (e.g., gefitinib, erlotinib, afatinib, and dacomitinib) select for resistance due to the T790M point mutation (EGFR T790M) in 50% of patients [1–4]. In other words, before therapy, 50% of patients have pre-existing resistant T790M mutations. Only one or a few cells contain the T790M mutation, because this resistant mutation confers no selective advantage prior to therapy. As soon as treatment starts, these rare cells selectively proliferate and eventually produce billions of cells [5], rendering the tumor resistant to the 1st/2nd generation of tyrosine kinase inhibitors (TKI). The cell with T790M is sensitive to the 3rd generation TKI osimertinib.

In 2015, the FDA approved osimertinib for NSCLC with a T790M mutation as a second-line therapy. This approval as a second-line therapy was based on the understanding that an untreated tumor cannot be T790M-positive; the mutation may initially occur in only one cell. Furthermore, 50% of patients do not harbor this mutation. It might seem logical to administer osimertinib after a tumor develops resistance to first- or second-generation TKIs, given the uncertainty regarding which patients will acquire the T790M mutation.

Consider a hypothetical scenario: If osimertinib were a combination of two medications (the first targets oncogenic EGFR without secondary T790M and the second targets only oncogenic EGFR with secondary T790M), then an oncologist would not prescribe the second drug to a patient sensitive to the first drug. And why would an oncologist prescribe the second (anti-resistant) drug? The tumor is not resistant to the first drug. Even if a patient has a single cell with T790M, its killing will be unnoticed at first, even in the months. The response rate will not be affected too. But after a year, even a transient addition of the anti-T790M drug at the beginning of treatment would prevent acquiring the resistance by the entire tumor and dramatically extend progression-free survival (PFS) and overall survival (OS). Osimertinib should be administered without waiting for the tumor to develop resistance, as it is feasible to eliminate a T790M-positive cell, but impossible to eradicate all million cells once the mutation is widespread. The latter scenario fails because of tumor heterogeneity, bad luck, and a mere probability. If the probability to kill one cell out of one is 0.99, then the probability to kill 2 cells out of 2 is 0.99 × 0.99 = 0.98, and the probability of killing all million cells is practically zero.

### Preemptive two-drug combinations

**Figure 1:**
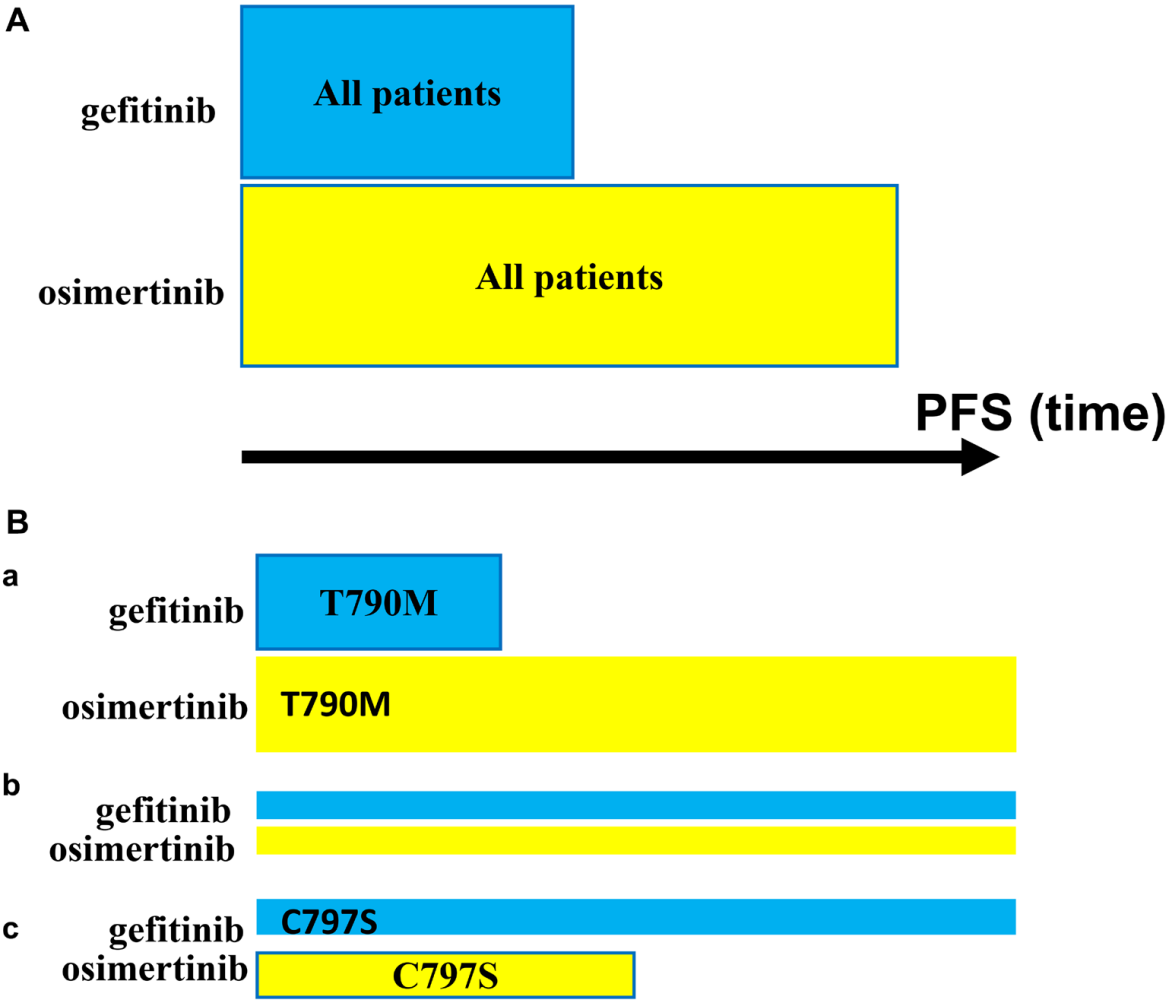
Progression-free survival (PFS): Gefitinib vs. Osimertinib. (**A**) PFS in a Total Cohort with EGFR-Mutant-Driven NSCLC: Patients Treated with Gefitinib vs. Osimertinib. (**B**) PFS in Cohorts Depending on Pre-existing Secondary Mutation within EGFR-Mutant: T790M vs. C797S. (**a**) T790M. (**b**) Neither T790M nor C797S. (**c**) C797S. To simplify, other pre-existing alterations are not shown.

Here I will re-introduce the notion of preemptive combinations (PC) of targeted drugs. Such combinations include the therapeutic response activities and anti-resistant activities that eliminate a few resistant cells. In other words, preemptive combinations induce both a therapeutic response and eliminate a few resistant cells with pre-existing mutations [6]. For example, if we started treatment with osimertinib and the tumor may have one cell with the C797S mutation (resistant to osimertinib but sensitive to gefitinib), then the addition of gefitinib to osimertinib renders this combination preemptive. The goal is to eliminate this one resistant cell. And vice versa, if we treat the tumor with gefitinib and this tumor may have a single cell with the T790M mutation (resistant to gefitinib but sensitive to osimertinib), then the addition of osimertinib to gefitinib creates the preemptive combination. Figuratively, osimertinib is equivalent to a preemptive combination of two activities: one activity causes tumor shrinkage (by targeting billions of cells without T790M and the second activity just to kill one cell with T790M. One cell is not noticeable. At first, its presence or absence does not affect the response rate and, if a response occurs, the degree of therapeutic response. Killing one resistant cell cannot increase the response rate and initial degree of response. But killing of this one cell (if a patient has it) extends PFS and OS. This must be done to prevent future resistance in 50% of patients and extend PFS. We should not wait for radiological progression or detection of T790M by biopsy to kill a resistant cell. One cell can be killed and all cells out of a billion cells cannot. The view on osimertinib as an analog of preemptive combination predicts the outcome of first-line treatment, when a tumor is not yet T790M-positive. The response rate will not be increased. In responders, the degree of initial response will not be changed. But PFS and OS will be extended in responders. That is exactly what was observed in a famous clinical trial published in 2018 [7]. The median progression-free survival (PFS) was significantly longer with osimertinib than with the 1st generation of EGFR inhibitors (18.9 months vs. 10.2 months; *P* < 0.001). The median duration of response was 17.2 months with osimertinib versus 8.5 months with 1st generation inhibitors. Yet, the objective response rate was similar in the two groups: 80% with osimertinib and 76% with 1st generation EGFR-TKIs [7]. Compared with the 1st generation of TKI, osimertinib prolongs PFS almost twofold (Figure 1A). This is especially dramatic given that not all patients have T790M. But osimertinib must be given to all patients because we do not know which patient has this mutation. If T790M were the only resistance mechanism, then PFS would be eternal, so patients would have a normal lifespan on chronic treatment with osimertinib. But roughly half of the resistance mechanisms are on-target such as secondary L718, G724, L792, G796, C797 in EGFR, and all off-target mechanisms such as MET and HER2 amplifications. Osimertinib selects for on-target resistance due to secondary mutations such as L718, G724, L792, G796, C797 [1, 8, 9] and the cells with these mutations can be targeted by the 1st or/and 2nd generation of EGFR inhibitors such as gefitinib and afatinib. So, addition of these EGFR inhibitors to osimertinib would prevent on-target resistance.

### Preemptive multi-drug combinations

The most common off-target resistances acquired after treatment with EGFR inhibitors are amplification of MET and HER2/3/4, and the latter is sensitive to afatinib. Adding capmatinib (the most effective MET inhibitor) would prevent MET-dependent resistance.

What percentage of patients with EGFR-driven cancer would benefit from a preemptive combination like Osimertinib, afatinib, and capmatinib?

Monotherapy with 1st or 2nd-generation EGFR TKIs causes resistance in 75% of patients due to either secondary T790M EGFR mutation plus MET or HER2 amplification [2–4, 10].

Monotherapy with 3rd-generation EGFR-TKI (osimertinib) causes at least 50% of resistance cases due to secondary mutations such as L718, G724, L792, G796, C797 plus MET and HER2 amplification [1, 9]. Specifically, resistance mechanisms to second-line osimertinib include MET 5–50%; HER2 5%; on-target 10–26% (mostly C797X); KRAS 2–8% [9]. Resistance mechanisms to first-line osimertinib include MET 7–15%; HER2 1–2%; on-target 6–10% (mostly C797X); KRAS 3–4% [9].

Thus, we can calculate that at least 75% of resistance mechanisms are preventable by a combination of osimertinib, afatinib, and capmatinib (OAC). Whether the combination should include afatinib or a 1st-generation TKI may depend on the primary activating mutation in the EGFR: del19 or L858R or uncommon. Osimertinib, afatinib, capmatinib (OAC) is most preferable because afatinib (i) inhibits HER2-4 [11] (ii) shows improved PFS compared with 1st generation [12, 13], especially when followed by osimertinib [14], and is effective in targeting brain metastases [11].

I estimate that by preventing 75% of resistance mechanisms, a combination of osimertinib, afatinib, and capmatinib (OAC) would extend median PFS from 18 months (osimertinib alone) to approximately 40 months. Such a remarkable extension takes into account that 25% of patients cannot benefit from OAC because they do not have pre-existing mechanisms of relevant resistance. However, all EGFR-dependent patients must be treated because it is not clear who will benefit. It is a mistake to wait for tumor progression or for resistance detection by biopsy.

### Even superior monotherapy hurts 20% patients

**Figure 2:**
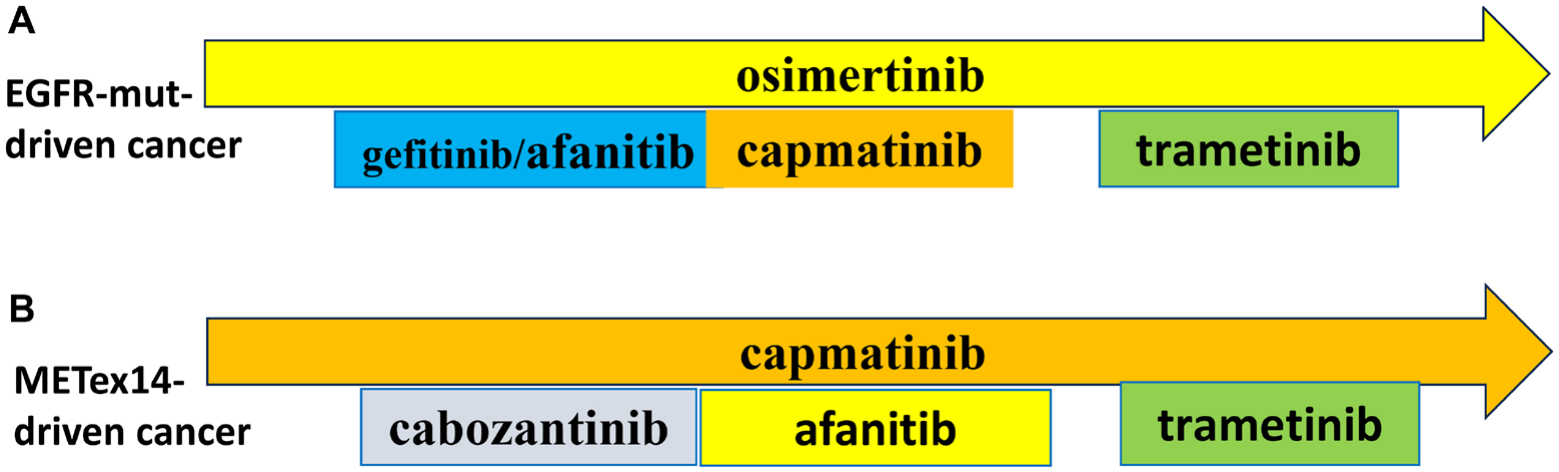
Preemptive combinations. (**A**) EGFR-Mutant-Driven NSCLC. See text. (**B**) METex14-Driven NSCLC. See text.

Osimertinib prolongs median progression-free survival (PFS) and overall survival (OS) compared with first-generation tyrosine kinase inhibitors (TKIs), such as gefitinib. Osimertinib is superior because the osimertinib-sensitive mutation (T790M) is more common than osimertinib-resistant mutations, such as C797S. We may suggest that osimertinib extends median PFS by prolonging PFS only in patients who have the T790M mutation (Figure 1B (a)). In patients lacking the T790M mutation, osimertinib should not extend PFS beyond the extension afforded by first-generation TKIs such as gefitinib (Figure 1B (b)). Furthermore, in a patient lacking T790M and having a preexisting C797S mutation, osimertinib selects for C797S (and L718, G724, L792, G796), making the tumor resistant and thus shortening PFS compared to first-generation TKIs such as gefinitib (Figure 1B (c)). Therefore, although most patients (approximately 55%) benefit from osimertinib, some patients (approximately 20%) are harmed. It is impossible to detect one (or a few) cells with a resistant mutation in untreated patients, so we must choose osimertinib for all patients. If we are obligated to use monotherapy, many patients (approximately 20%) will be adversely affected, even though the median PFS for the entire cohort is improved. Neither the unfortunate patient nor patient’s doctor can know that superior TKI shortened PFS in this particular patient. The solution is remarkably simple: to use preemptive combinations of osimertinib + gefitinib (O+G) and osimertinib + gefitinib + afatinib (O+G+A) from the start. Current therapy drives resistance and even finds a way to achieve the infamous cis-T790M/C797S triple mutations that are resistant to all existing EGFR inhibitors and any of their combinations [15]. This cis mutation will not appear when the O+G combination is used preemptively. What is more, there will be no need for the development of a fourth generation of EGFR inhibitors to target this cis mutation. In addition, preemptive combinations should include drugs that target the most common off-target mechanisms, such as MET inhibitors (capmatinib). Preemptive combinations can be used in a sequence of transient two-drug combinations (Figure 2A) if an oncologist is uncomfortable with three- and four-drug combinations. (Ironically, an oncologist is comfortable with multi-kinase inhibitors such as lenvatinib and cabozantinib against one intended target, which is akin to a multi-drug combo with one intended target and all other random drugs. If so, why then an oncologist is uncomfortable with preemptive combinations of selective inhibitors). Preemptive combinations to prevent both on-target and off-target resistance have been discussed [6]. In general, combinations are necessary to abrogate resistance [10, 16–20].

If a patient has EGFR-driven NSCLC, they are in some ways fortunate because this is the most common type of lung cancer, and it has been studied extensively. There are three generations of inhibitors available, and drug combinations, especially with MET inhibitors, are occasionally used. The treatment for MET-driven lung cancer is less developed, but the insights gained from EGFR-driven cancer can be beneficial. The approaches to treatment can be similar.

### Preemptive combination for MET-driven NSCLC

A year ago, I was hospitalized at Massachusetts General Hospital (Boston) with multiple brain metastases from lung cancer, driven by a MET exon 14 skipping mutation (METex14) [6]. The response to capmatinib (a selective MET inhibitor, type I) was outstanding. However, resistance tends to develop within a year of treatment [21]. The most common mechanism of on-target resistance involves secondary mutations in METex14, such as D1228 and Y1230 [22]. Resistance mutations against type I MET inhibitors are sensitive to type II inhibitors, and vice versa [22–26]. In patients with METex14 NSCLC, cabozantinib can overcome resistance selected by type I MET inhibitors [27–29]. Furthermore, cabozantinib is effective in METex14-positive NSCLC with brain metastases [30].

Cabozantinib is the only FDA-approved type II MET inhibitor, so our choices are limited to this multi-kinase inhibitor, which has unpleasant side effects if used long-term. Yet, cabozantinib can be used transiently for a few weeks, for example, to eliminate cells with pre-existing mutations such as D1228 or Y1230 before they expand due to capmatinib. Simultaneous treatment with type I and type II MET inhibitors may delay the emergence of on-target MET resistance mutations [31].

The most common off-target mechanisms of resistance include alterations in EGFR and HER2 [26, 32, 33]. These can be targeted by afatinib (Figure 2B). Combinations including a MEK inhibitor (trametinib) will be discussed in the forthcoming article *“My Battle with Cancer: Part III.”*

Preemptive treatment with capmatinib, afatinib, and cabozantinib may prevent 50% of all potential resistance, and half of all patients with METex14-driven lung cancer may experience prolonged progression-free survival (PFS), leading to a longer and happier life.
